# Supplementary material for: CMTM4 is a subunit of the IL-17 receptor and mediates autoimmune pathology
Source: Nat Immunol. 2022 Oct 21;23(11):1644–52. doi: 10.1038/s41590-022-01325-9 (PMC9663306; doi:10.1038/s41590-022-01325-9)

nature immunology

Article

https://doi.org/10.1038/s41590-022-01325-9

CMTM4 is a subunit of the IL-17 receptor and mediates autoimmune pathology

In the format provided by the authors and unedited

Fig. 5a - Gating strategy

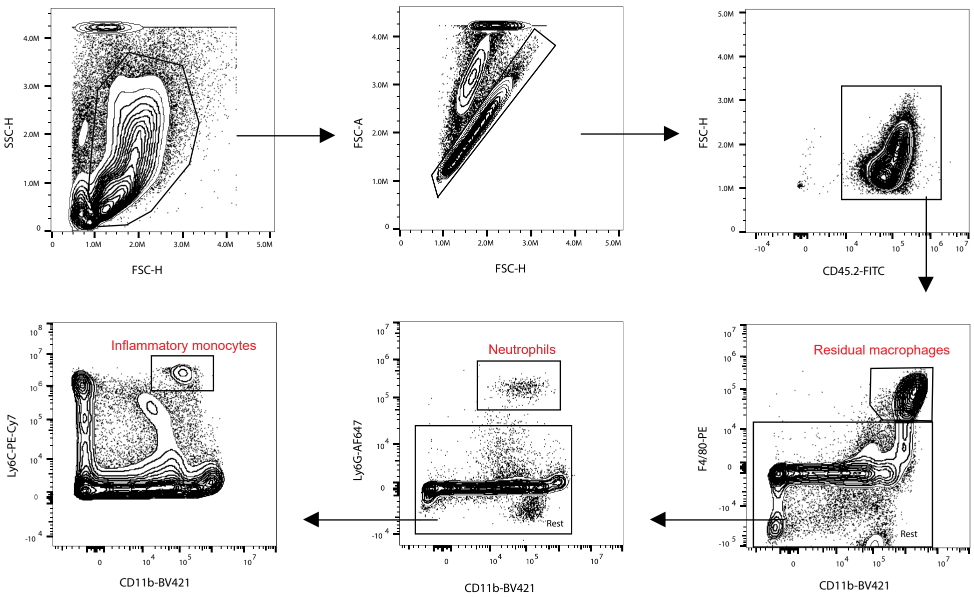

Fig. 7d - Gating strategy

B cells

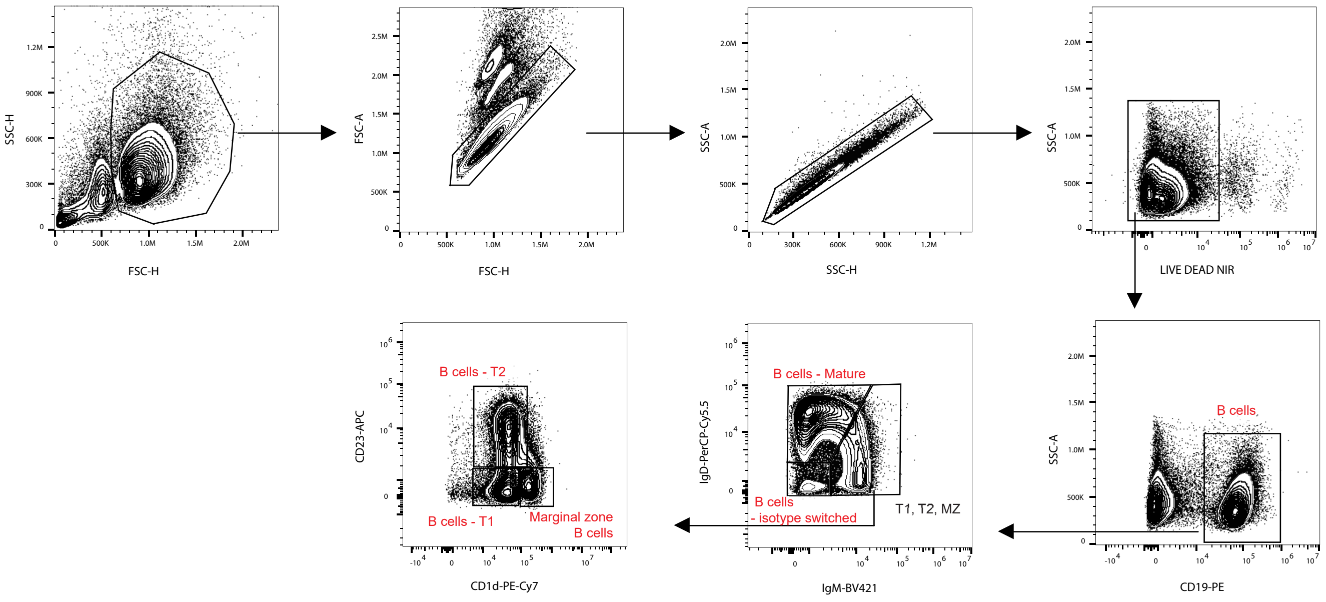

Monocytes - Macrophages

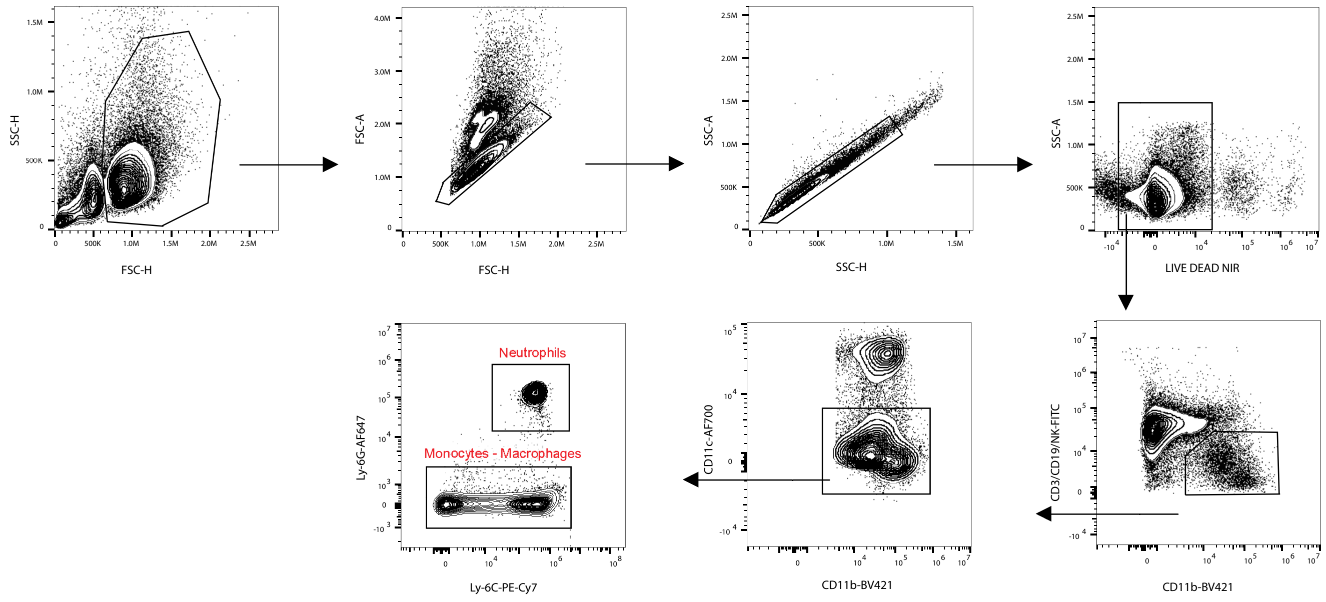

Fig. 7d - Gating strategy

CD8^{+} T cells

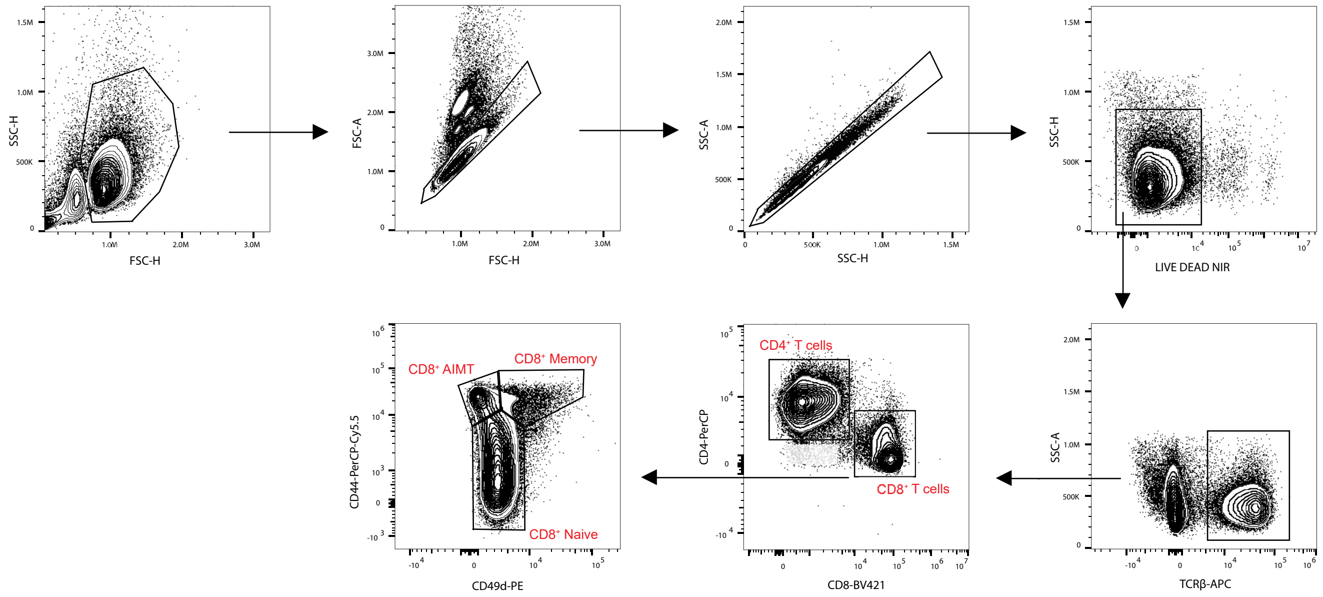

CD4^{+} T cells

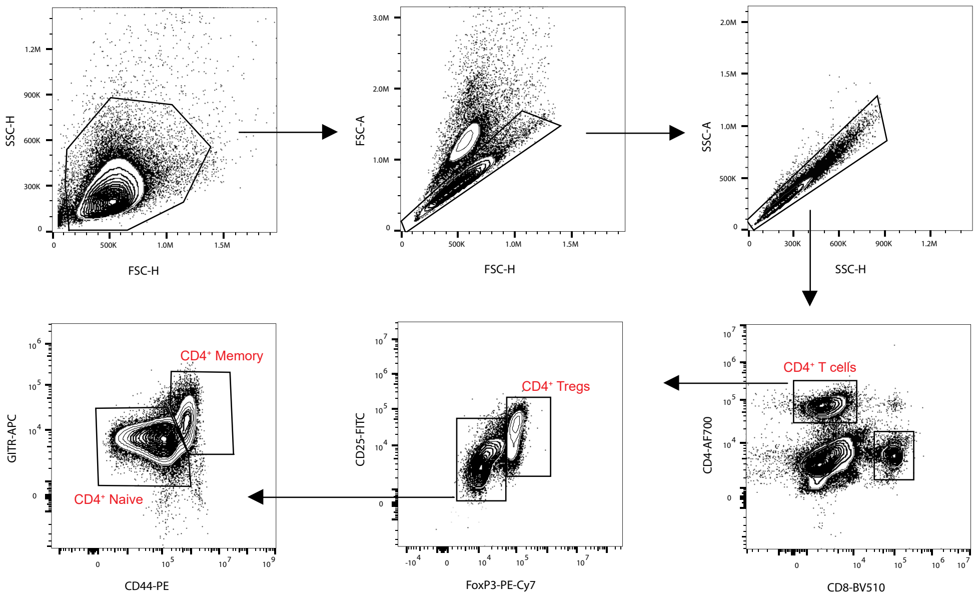

Supplement: Supplementary file 1 — Supplementary Information [file 41590_2022_1325_MOESM1_ESM.pdf]
