# Supplementary material for: CMTM4 is a subunit of the IL-17 receptor and mediates autoimmune pathology
Source: Nat Immunol. 2022 Oct 21;23(11):1644–52. doi: 10.1038/s41590-022-01325-9 (PMC9663306; doi:10.1038/s41590-022-01325-9)
Supplement: Supplementary file 2 — Reporting Summary [file 41590_2022_1325_MOESM2_ESM.pdf]

nature research

Corresponding author(s): Peter Draber and Ondrej Stepanek

Last updated by author(s): Aug 30, 2022

Reporting Summary

Nature Research wishes to improve the reproducibility of the work that we publish. This form provides structure for consistency and transparency in reporting. For further information on Nature Research policies, see our Editorial Policies and the Editorial Policy Checklist.

Statistics

For all statistical analyses, confirm that the following items are present in the figure legend, table legend, main text, or Methods section.

n/a	Confirmed	
☐	☒	The exact sample size (n) for each experimental group/condition, given as a discrete number and unit of measurement
☐	☒	A statement on whether measurements were taken from distinct samples or whether the same sample was measured repeatedly
☐	☒	The statistical test(s) used AND whether they are one- or two-sided<br>*Only common tests should be described solely by name; describe more complex techniques in the Methods section.*
☐	☒	A description of all covariates tested
☐	☒	A description of any assumptions or corrections, such as tests of normality and adjustment for multiple comparisons
☐	☒	A full description of the statistical parameters including central tendency (e.g. means) or other basic estimates (e.g. regression coefficient) AND variation (e.g. standard deviation) or associated estimates of uncertainty (e.g. confidence intervals)
☐	☒	For null hypothesis testing, the test statistic (e.g. F, t, r) with confidence intervals, effect sizes, degrees of freedom and P value noted<br>*Give P values as exact values whenever suitable.*
☒	☐	For Bayesian analysis, information on the choice of priors and Markov chain Monte Carlo settings
☒	☐	For hierarchical and complex designs, identification of the appropriate level for tests and full reporting of outcomes
☒	☐	Estimates of effect sizes (e.g. Cohen's d, Pearson's r), indicating how they were calculated

Our web collection on statistics for biologists contains articles on many of the points above.

Software and code

Policy information about availability of computer code

Data collection	*Provide a description of all commercial, open source and custom code used to collect the data in this study, specifying the version used OR state that no software was used.*
Data analysis	All MS data were analyzed and quantified with the MaxQuant software (version 1.6.5.0) and subsequent data analysis was performed using Perseus 1.6.14.0 software. Cytometry data were analyzed using FlowJo software (TreeStar).

For manuscripts utilizing custom algorithms or software that are central to the research but not yet described in published literature, software must be made available to editors and reviewers. We strongly encourage code deposition in a community repository (e.g. GitHub). See the Nature Research guidelines for submitting code & software for further information.

Data

Policy information about availability of data

All manuscripts must include a data availability statement. This statement should provide the following information, where applicable:

- Accession codes, unique identifiers, or web links for publicly available datasets
- A list of figures that have associated raw data
- A description of any restrictions on data availability

The MS proteomics data have been deposited to the ProteomeXchange Consortium via the PRIDE under accession number PXD036366. RNASeq data were downloaded from Gene Expression Omnibus under accession numbers GSE159929, GSE130973, GSE54456, GSE121212. Source data are provided with this paper for all figures.

Field-specific reporting

Please select the one below that is the best fit for your research. If you are not sure, read the appropriate sections before making your selection.

☒ Life sciences ☐ Behavioural & social sciences ☐ Ecological, evolutionary & environmental sciences

For a reference copy of the document with all sections, see nature.com/documents/nr-reporting-summary-flat.pdf

Life sciences study design

All studies must disclose on these points even when the disclosure is negative.

Sample size

All experiments were repeated several times as specified in the manuscript. In the case of graphs, the experiments were repeated at least 5 times to allow statistical analysis via Mann-Whitney test or t test the normality of data via Kolmogorov-Smirnov test. Alternatively for experimental results that were assumed to follow normal distribution, three repetitions were performed and statistical significance was calculated using Student's t-test. In the case of mice cohorts, generally accepted sample size were used based on prior experience with the model.

Data exclusions

No data were excluded from analyses.

Replication

The number of replication for each experiment is specified in each figure. All experiments were reliably reproduced. In particular, we confirmed all our findings in cell lines of both human and mouse origin.

Randomization

Mice were allocated to particular test groups based on their genotype. Littermates expressing or not CMTM4 were compared in blind manner. Experiment using cell lines were not randomized, the design of individual experiments allowed to control for covariants.

Blinding

In order to assess the increase of thickness and scaling during experimental psoriasis, mice were randomly distributed in cages prior to the experiment. Mice were photographed daily in a blinded manner to their genotype. Photographs were subsequently analyzed in a blinded manner in random order. In order to assess the severity of EAE, mice were randomly distributed in cages prior to the experiment. The induction of EAE and daily observation of the disease severity were performed in a blinded manner. In the case of experiments using cell lines, the blinding was not performed as we compared different cell lines side by side.

Reporting for specific materials, systems and methods

We require information from authors about some types of materials, experimental systems and methods used in many studies. Here, indicate whether each material, system or method listed is relevant to your study. If you are not sure if a list item applies to your research, read the appropriate section before selecting a response.

Materials & experimental systems

n/a	Involved in the study	
☐	☒	Antibodies
☐	☒	Eukaryotic cell lines
☒	☐	Palaeontology and archaeology
☐	☒	Animals and other organisms
☒	☐	Human research participants
☒	☐	Clinical data
☒	☐	Dual use research of concern

Methods

n/a	Involved in the study	
☒	☐	ChIP-seq
☐	☒	Flow cytometry
☒	☐	MRI-based neuroimaging

Antibodies

Antibodies used

Antibodies detecting β-Actin (Cat. number 3700), IκBα (9242), P-p105 (Ser932) (4806), P-p38 (Thr180/Tyr182) (9216), P-JNK (Thr183/Tyr185) (4671), P-TBK1(S172) (5483), Myc (2276), TBK1 (3013), JNK2 (9258), p105 (4717 or 13586) were purchased from Cell Signaling Technology; CMTM4 (HPA014704), FLAG (F3165), GFP (SAB4301138), human IL-17RC (HPA019885) were from Sigma-Aldrich; ACT1 (sc-398161), and p38 (sc-728) was from Santa Cruz Biotechnology; murine IL-17RA (MAB4481), IL-17RC (AF2270), IL-17RD (AF2276), and TNFR1 (AF-425-PB) were from R&D Systems; TRAF6 (ab40675) was from Abcam. Standard dillution of antibodies for immunoblotting was 1:1000.
Secondary antibodies Anti-Rabbit IgG (H+L) HRP (711-035-152), Goat Anti-Mouse IgG1 HRP (115-035-205), Goat Anti-Mouse IgG2a HRP (115-035-206), Goat Anti-Mouse IgG2b HRP (115-035-207), Donkey anti-Goat IgG H+L HRP (705-035-147), Goat Anti-Rabbit IgG Fc fragment specific HRP (111-035-046), Goat Anti-Rat IgG (H+L) HRP (112-035-167), Donkey Anti-Rabbit IgG (H+L) AF488 (711-545-152), Donkey Anti-Goat IgG (H+L) AF647 (705-605-147) were purchased from Jackson Immunoresearch, dilution 1:10000 for HRP conjugated antibodies, 1:500 for fluorophore conjugated antibodies. For detection of ACT1, we used Mouse TrueBlot ULTRA (Rockland), dilution 1:5000.
Fluorescently labelled antibodies against following antigens were used: CD1d Pe-Cy7 (123524), CD4 PerCP (100538), CD8a BV421 (100738), CD8a BV510 (100752), CD11b BV421 (101251), CD11c AF700 (117320), CD19 PE (115508), CD25 PE-Cy7 (102016), CD44 PE (103008), CD44 PerCP-Cy5.5 (103032), GITR APC (126312), F4/80 PE (123110), IgM BV421 (406518), IgD PerCP-Cy5.5 (405710),

KLRG1 BV510 (138421), Ly6C PE-Cy7 (128018), Ly6G AF647 (127610), TCRβ PerCP (109212), MCP-1/CCL2 PE (505903), B220 AF700 (103232), TCRβ PE (109208), CD45.1 BV650 (110735), and human IL-17RA-APC (372305) were purchased from BioLegend; CD3 FITC (553062), CD19 FITC (553785), CD25 FITC (553071), CD45.2 FITC (553772), CD49d PE (553157), and NK-1.1 FITC (553164) were from BD Pharmingen; CD23 APC (1108095) was from SONY; FOXP3 PE-Cy7 (25-5773-82) was from eBioscience, Calnexin AF647 (MA3-027-A647) was from Invitrogen. Standard dilution of fluorescently conjugated antibodies was 1:200. LIVE/DEAD Fixable Near-IR Dead Cell Stain Kit was from Thermo Fisher Scientific.

Validation

Most antibodies used in this study are commonly used by us and other laboratories. The validation of individual antibodies can be find on manufacturer's website. CMTM4, IL-17RC, Act1, TRAF6 antibodies were validated in knockout cell lines.

Eukaryotic cell lines

Policy information about cell lines

Cell line source(s)

ST2 cells were kindly provided by Jana Balounova, HeLa, HEK293FT, Phoenix-Eco and Phoenix-Ampho were kindly provided by Tomas Brdicka, and S2 cells were kindly provided by Petr Draber (all from Institute of Molecular Genetics, Prague, Czech Republic). A549 cells were kindly provided by Zora Novakova (Institute of Biotechnology, Vestec, Czech Republic). These cell lines are also commercially available. MEF cells were derived from E11.5 mouse embryos and immortalized by lentiviral infection with the SV40 large T antigen.

Authentication

Listed cells are commonly used in our lab, no additional authentication was performed.

Mycoplasma contamination

All cell lines were regularly tested for mycoplasma contamination via PCR and were mycoplasma negative.

Commonly misidentified lines
(See ICLAC register)

No commonly misidentified cell lines were used in the study.

Animals and other organisms

Policy information about studies involving animals; ARRIVE guidelines recommended for reporting animal research

Laboratory animals

Frozen sperm from mouse strain C57BL/6N-Atm1Brd Cmtm4tm1a(EUCOMM)Wtsi/WtsiBiat was obtained from The European Mouse Mutant Archive repository (EM:06038) and used for in vitro fertilization. Mice carrying targeted Cmtm4 allele were crossed with Flp-deleter mouse strain B6.Cg-Tg(ACTFLPe)9205Dym/J from The Jackson Laboratory (005703). The resulting mouse strain with exon 2 and 3 flanked by LoxP sites was crossed with Cre-deleter strain Gt(ROSA)26Sortm1(ACTB-cre,-EGFP)Ics (MGI:5285392, Philippe Soriano) to obtain germ line knockout mouse. Animals were kept on C57BL/6J background. All mice used in experiments were 5-12 weeks old. TThey were housed in specific pathogen-free facility 12h/12h light/dark cycle, temperature and relative humidity are maintained at 22 ± 1 °C and 55 ± 5 %, respectively. Both males and females were used for experiments, except for IMQ-induced psoriasis and EAE where female littermate mice were analyzed. If possible, littermates were equally divided into the experimental groups.

Wild animals

Study did not involve wild animals.

Field-collected samples

Study did not involve samples collected from field.

Ethics oversight

Animal protocols were approved by the Resort Professional Commission for Approval of Projects of Experiments on Animals of the Czech Academy of Sciences, Czech Republic.

Note that full information on the approval of the study protocol must also be provided in the manuscript.

Flow Cytometry

Plots

Confirm that:

☒ The axis labels state the marker and fluorochrome used (e.g. CD4-FITC).

☒ The axis scales are clearly visible. Include numbers along axes only for bottom left plot of group (a 'group' is an analysis of identical markers).

☒ All plots are contour plots with outliers or pseudocolor plots.

☒ A numerical value for number of cells or percentage (with statistics) is provided.

Methodology

Sample preparation

In order to detect surface IL-17RC, cells were transferred to suspension, resuspended in FACS buffer (PBS, 2% FCS, 0.1% NaN_3) and stained with anti-IL17RC antibody on ice flowed by APC-labeled secondary antibody. Propidium iodide solution were used for discrimination of live and dead cells.

In order to detect production of CCL2, cells were washed with serum-free DMEM and stimulated for 4 hours with the indicated concentration of IL-17 in the presence of 5 μg/ml Brefeldin A (BioLegend). Cells were collected, fixed and

permeabilized using Cyto-Fast™ Fix/Perm Buffer Set (BioLegend) and stained with fluorescently labeled CCL2 antibody. Samples were measured on BriCyte E6 flow cytometer and data were analyzed using FlowJo software (TreeStar).

Peritoneal lavage was performed using 11 ml of PBS. Cell suspensions were stained with LIVE/DEAD near-IR dye (Life Technologies) and the mixture of primary antibodies (CD45.2, CD11b, F4/80, Ly6G, Ly6C) on ice and analyzed by flow cytometry using Cytek Aurora.

In order to analyze the immune cell populations in WT and CMTM4 KO mice, mice were sacrificed and the spleen, peripheral lymph nodes, and mesenteric lymph nodes were removed and single cell suspensions were prepared. In the case of spleen, red blood cells were lysed in ACK buffer (150 mM NH_4Cl, 10 mM $KHCO_3$, 0.1 mM EDTA- Na_2, pH 7.4). Cells were resuspended in FACS buffer and stained on ice with LIVE/DEAD near-IR dye (Life Technologies) and stained with ollowing sets of antibodies: T cell compartment (TCRβ, CD4, CD8, CD44, CD49d). B cell compartment (CD19, IgM, IgD, CD23, CD1d). Myeloid compartment (CD3, CD19, NK1.1, CD11b, CD11c, Ly6C, Ly6G). In order to distinguish Tregs and memory CD4 T cells, cells were fixed and permeabilized using Foxp3/Transcription Factor Staining Buffer Set (eBioscience, 00-5523-00) and stained with following antibodies (CD4, CD8, CD25, CD44, FoxP3, GITR).

Instrument	BriCyte E6 flow cytometer or Cytek Aurora
Software	FlowJo software (TreeStar)
Cell population abundance	Retrovirally transduced cells were sorted as GFP positive and the purity was regularly tested via FACS.
Gating strategy	Immune cell populations isolated from mice were gated as follows. T cells (TCRβ+) were separated in following subsets: CD4+ T cells (CD4+) and CD8+ T cells (CD8+), which were further divided in naïve CD8+ (CD44-), memory CD8 (CD44+, CD49d+), and antigen-inexperienced memory T cells (AIMT) (CD44+, CD49d-) cells. B cells (CD19+) were separated in following subsets: T1 (IgM+, CD23-, CD1d-), T2 (IgM+, CD23+, CD1d-), marginal zone B cells (IgM+, CD23-, CD1d+), mature (IgM-, IgD+), and isotype switched (IgM-, IgD-). Myeloid cells (CD3-, CD19-, NK1.1-, CD11b+) were separated in the following subsets: neutrophils (CD11c-, Ly6G+) and monocytes/macrophages (CD11c-, Ly6G-). CD4+ T cells were divided in subsets: Tregs (FoxP3+, CD25+) and CD4+ naïve cells (FoxP3-, CD44-) and CD4+ memory cells (FoxP3-, CD44+).Samples were measured on Cytek Aurora and data were analyzed using FlowJo software (TreeStar).<br><br>Cells isolated via peritoneal lavage, samples were first gated as CD45.2+ and subsequently separated in the following subsets: macrophages (CD11b+, F4/80+), neutrophils (CD11b+, Ly6G+) and inflammatory monocytes (CD11b+, Ly6G-, Ly6C+).

☒ Tick this box to confirm that a figure exemplifying the gating strategy is provided in the Supplementary Information.
