# Supplementary figures and images for: CMTM4 is a subunit of the IL-17 receptor and mediates autoimmune pathology
Source: Nat Immunol. 2022 Oct 21;23(11):1644–52. doi: 10.1038/s41590-022-01325-9 (PMC9663306; doi:10.1038/s41590-022-01325-9)

Fig. 1b

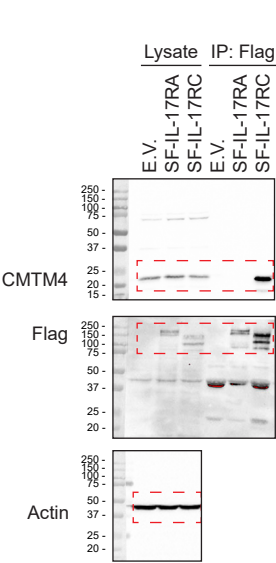

Fig. 1c

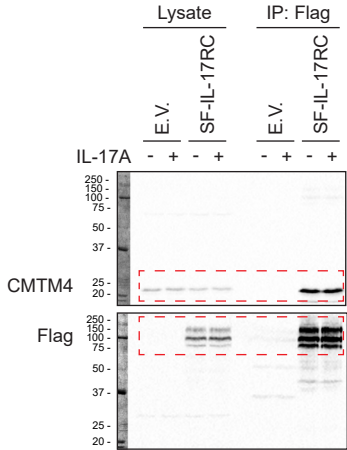

Fig. 1d

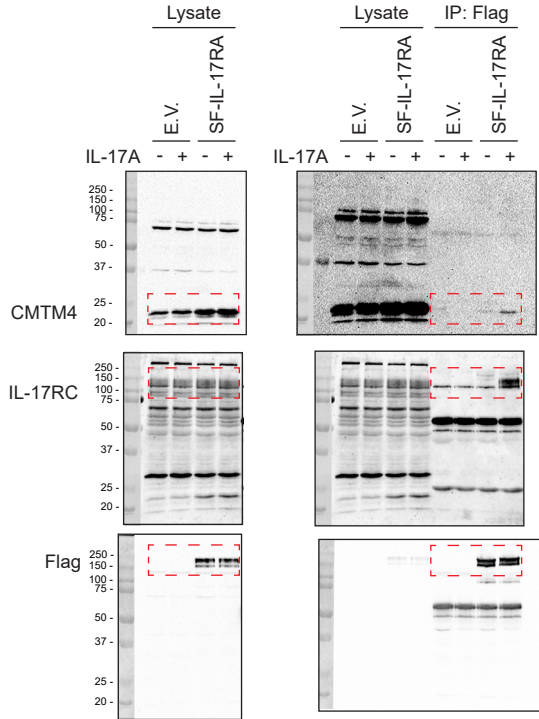

Fig. 1e

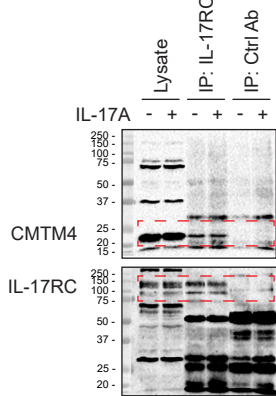

Fig. 1h

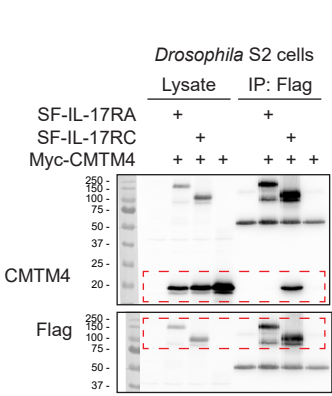

Fig. 1i

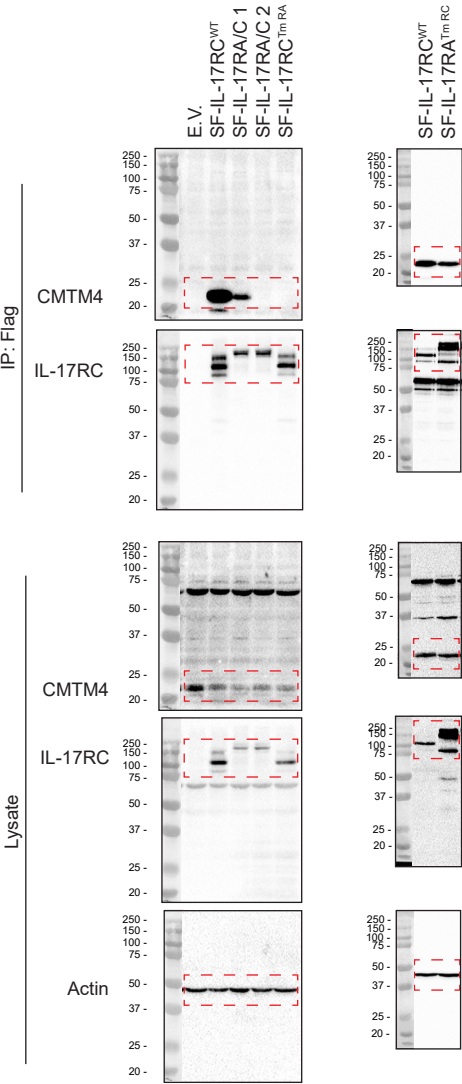

Fig. 1j

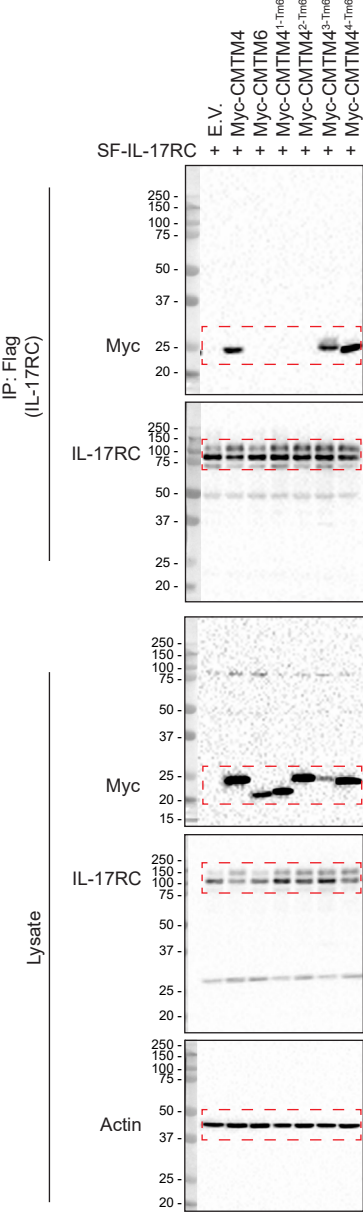

Supplement: Source Data Fig. 1 — Unprocessed western bots. [file 41590_2022_1325_MOESM5_ESM.pdf]

Fig. 2a

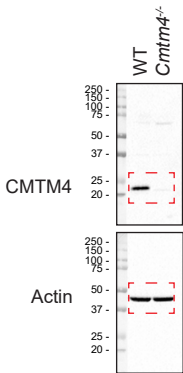

Fig. 2b

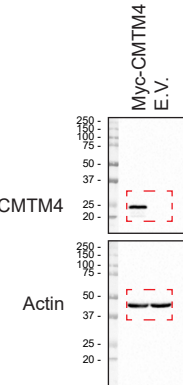

Fig. 2c

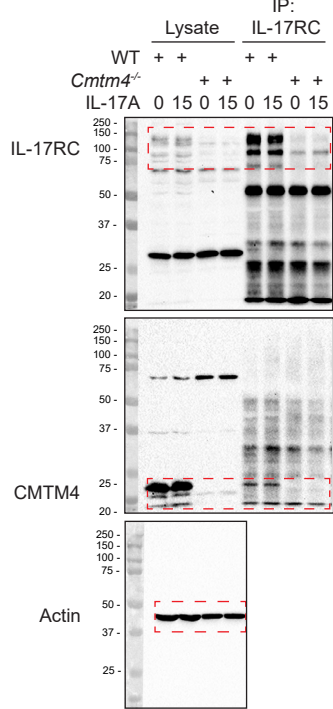

Fig. 2d

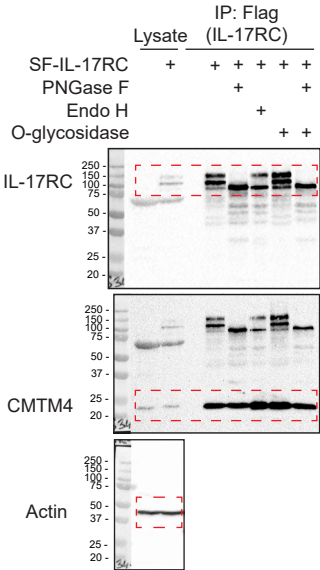

Fig. 2e

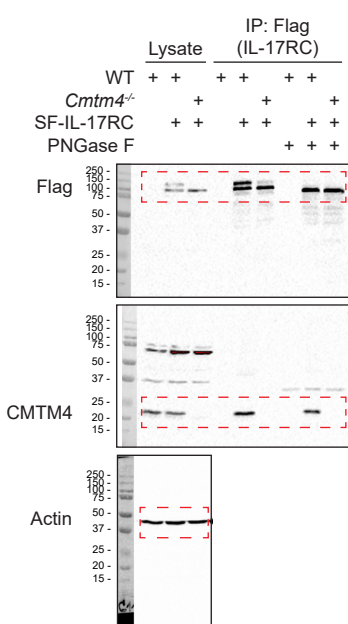

Fig. 2f

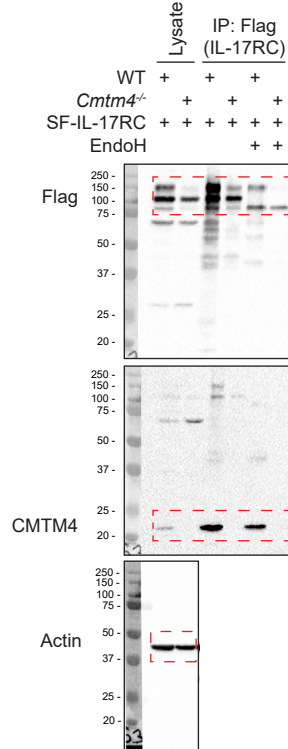

Supplement: Source Data Fig. 2 — Unprocessed western blots. [file 41590_2022_1325_MOESM6_ESM.pdf]

Fig. 3a

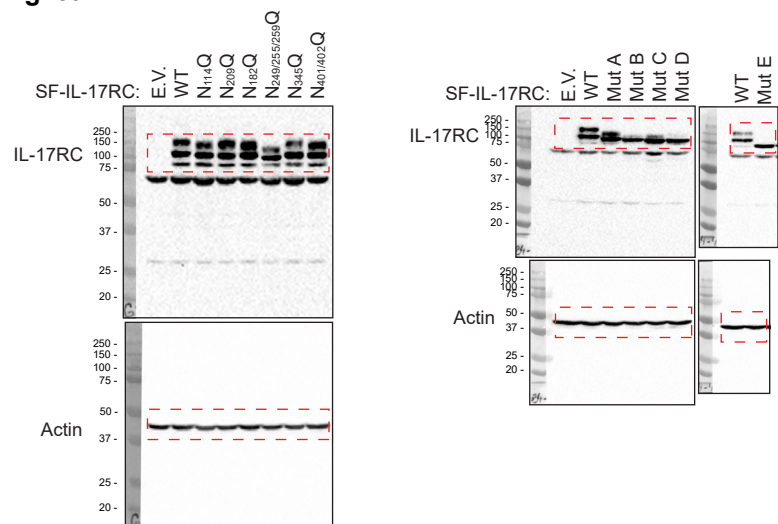

Fig. 3c

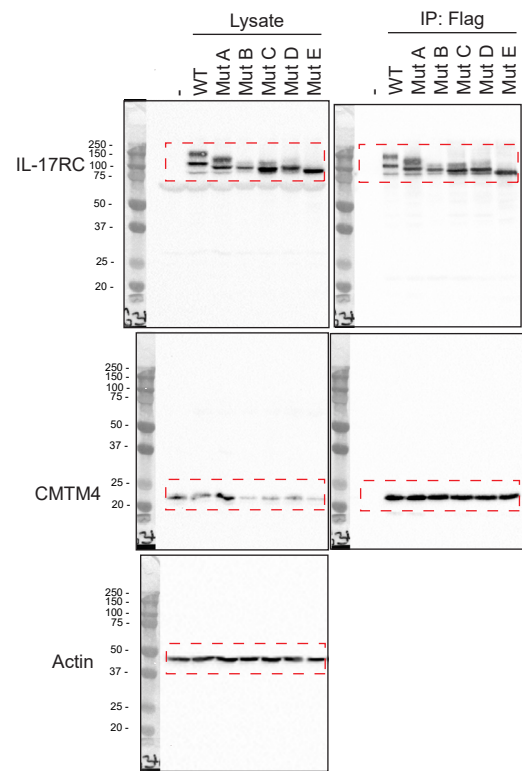

Fig. 3d

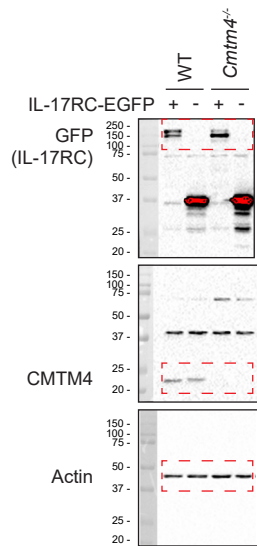

Fig. 3g

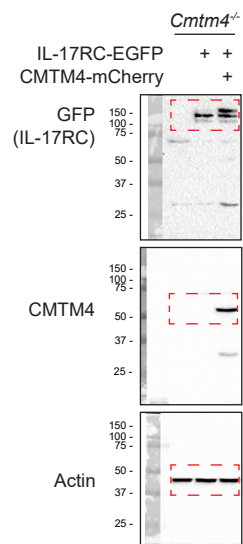

Supplement: Source Data Fig. 3 — Unprocessed western blots. [file 41590_2022_1325_MOESM8_ESM.pdf]

Fig. 4a

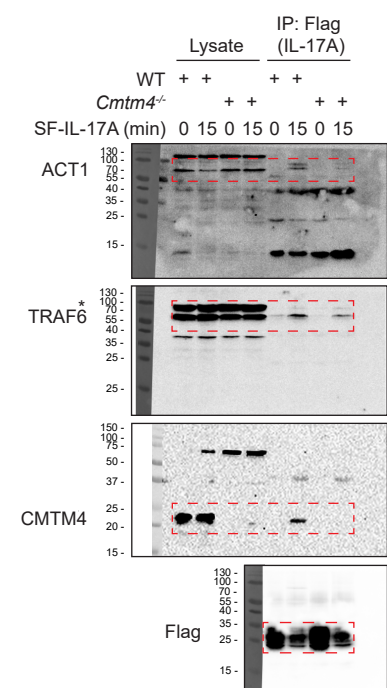

Fig. 4b

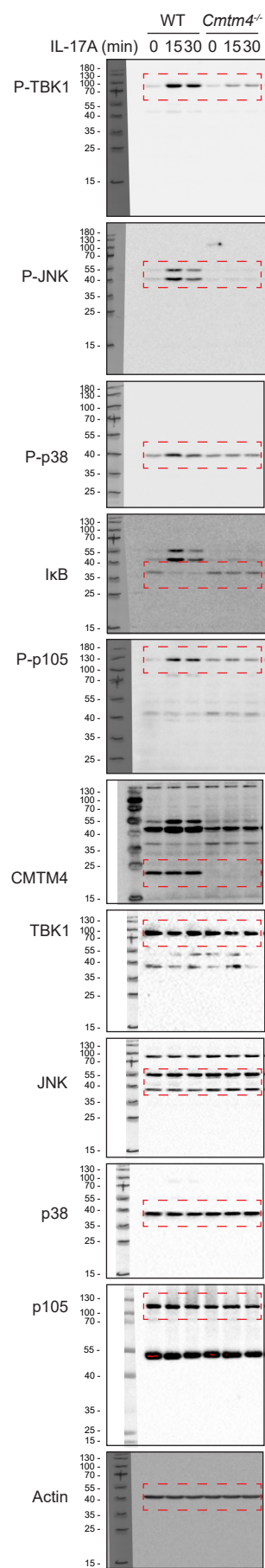

Fig. 4c

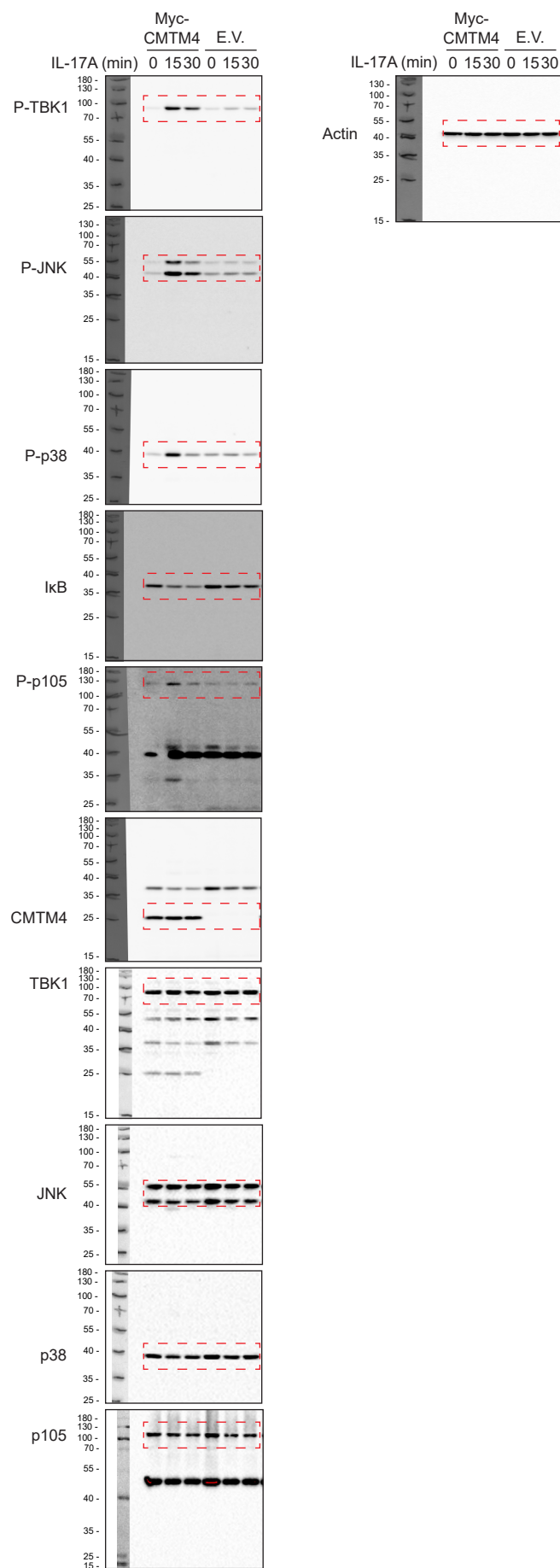

Supplement: Source Data Fig. 4 — Unprocessed western blots. [file 41590_2022_1325_MOESM10_ESM.pdf]

Extended Data Fig. 1c

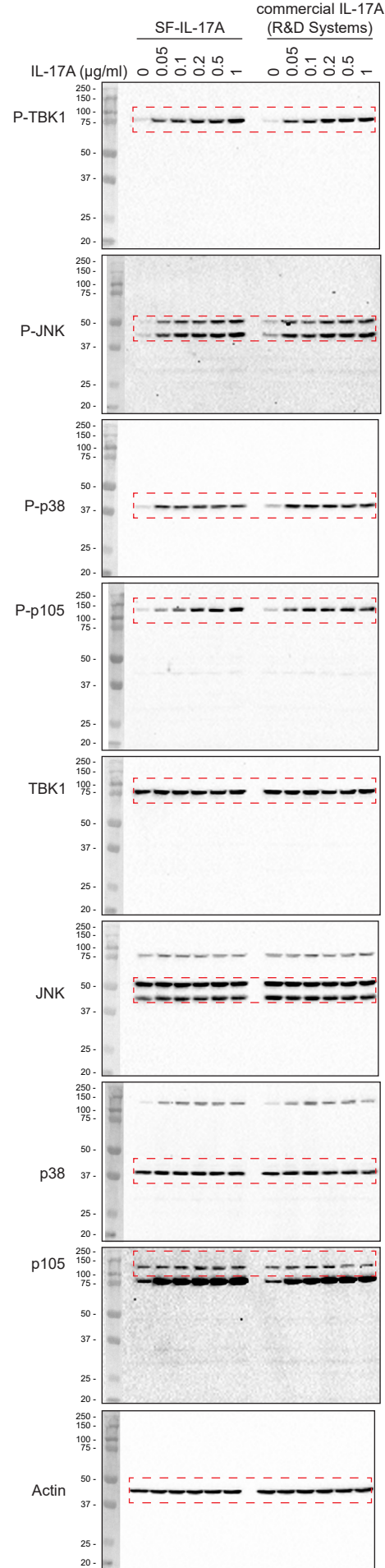

Extended Data Fig. 1d

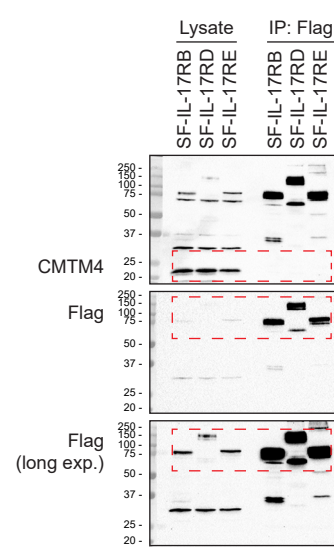

Supplement: Source Data Extended Data Fig. 1 — Unprocessed western blots. [file 41590_2022_1325_MOESM13_ESM.pdf]

Extended Data Fig. 7b

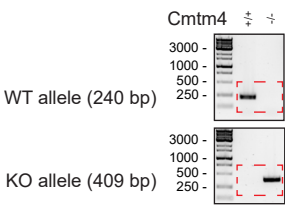

Extended Data Fig. 7e

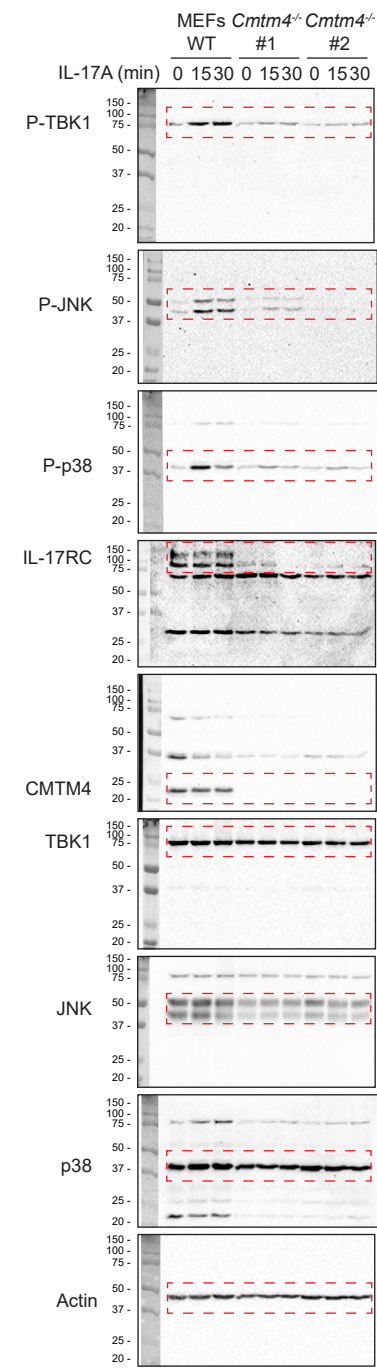

Supplement: Source Data Extended Data Fig. 7 — Unprocessed western blots. [file 41590_2022_1325_MOESM21_ESM.pdf]
