# Supplementary material for: CMTM4 is a subunit of the IL-17 receptor and mediates autoimmune pathology
Source: Nat Immunol. 2022 Oct 21;23(11):1644–52. doi: 10.1038/s41590-022-01325-9 (PMC9663306; doi:10.1038/s41590-022-01325-9)

Extended Data Fig. 2a

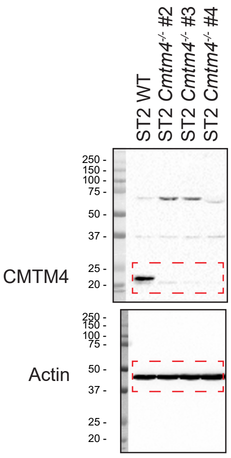

Extended Data Fig. 2b

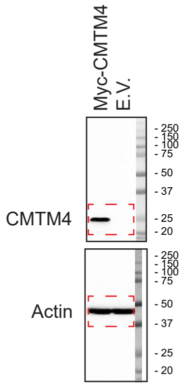

Extended Data Fig. 2e

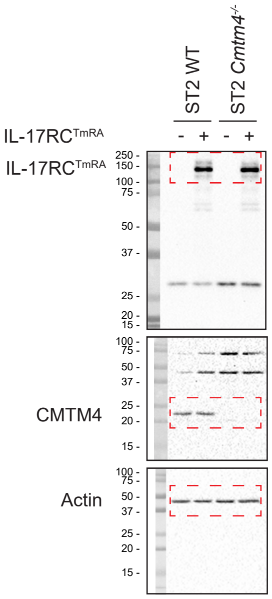

Extended Data Fig. 2f

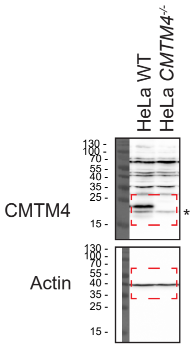

Ext. Data Fig. 2g

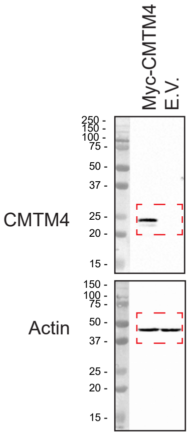

Extended Data Fig. 2h

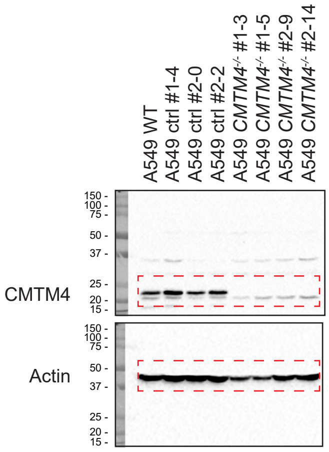

Supplement: Source Data Extended Data Fig. 2 — Unprocessed western blots. [file 41590_2022_1325_MOESM15_ESM.pdf]
