# Supplementary material for: CMTM4 is a subunit of the IL-17 receptor and mediates autoimmune pathology
Source: Nat Immunol. 2022 Oct 21;23(11):1644–52. doi: 10.1038/s41590-022-01325-9 (PMC9663306; doi:10.1038/s41590-022-01325-9)

Extended Data Fig. 3a

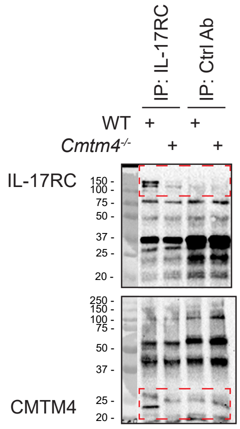

Extended Data Fig. 3b

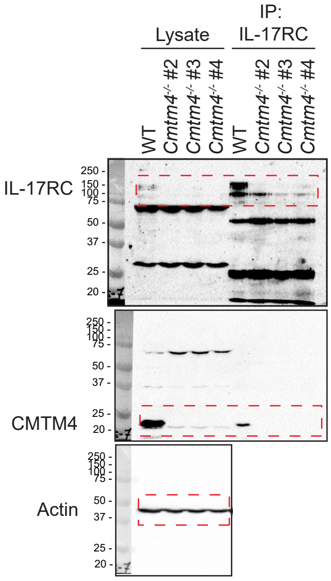

Extended Data Fig. 3c

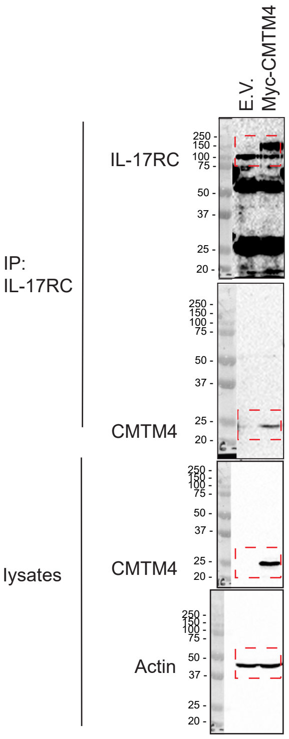

Extended Data Fig. 3d

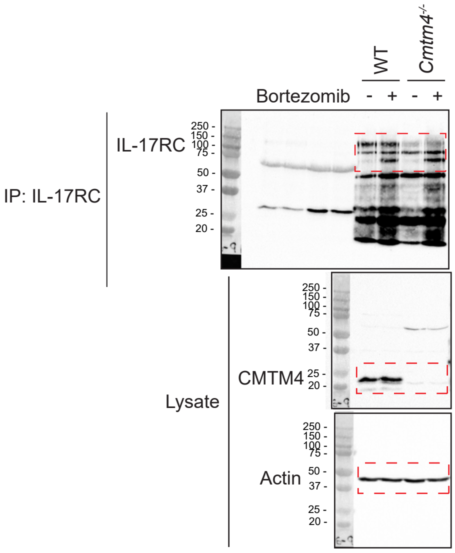

Extended Data Fig. 3e

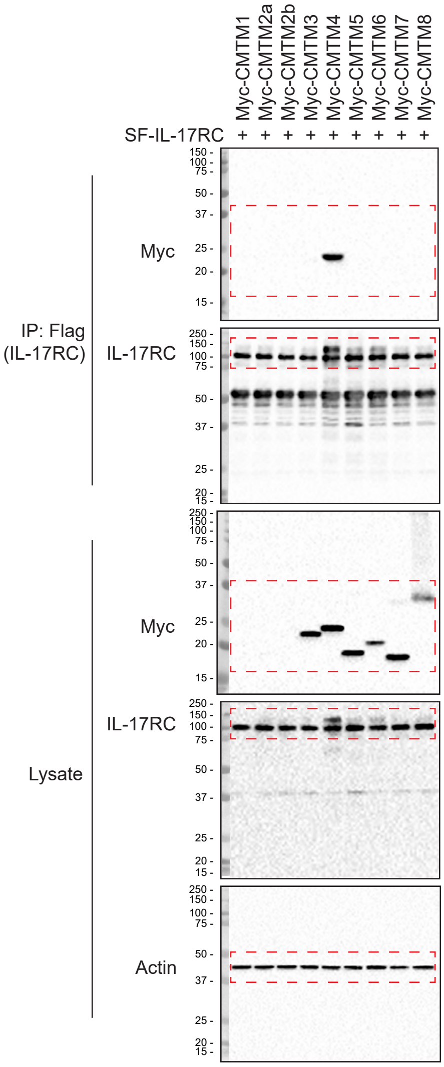

Extended Data Fig. 3f

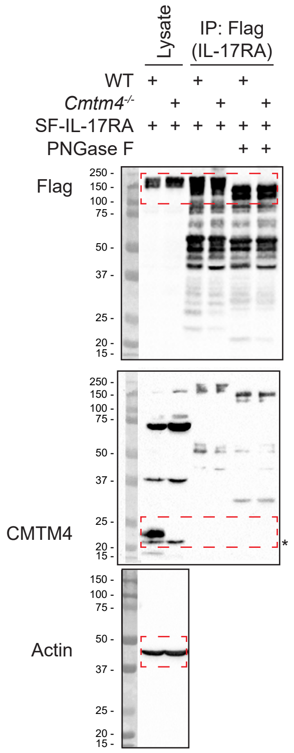

Extended Data Fig. 3g

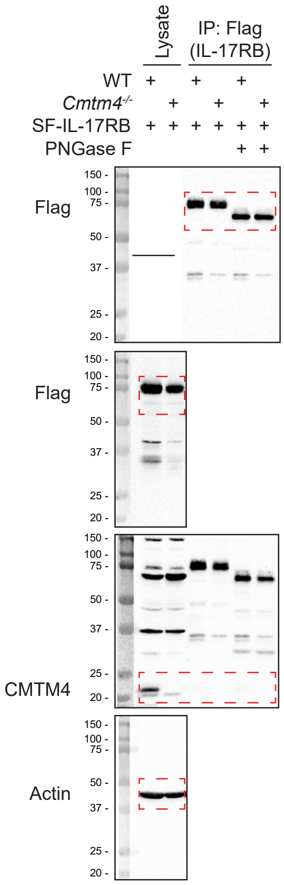

Extended Data Fig. 3h

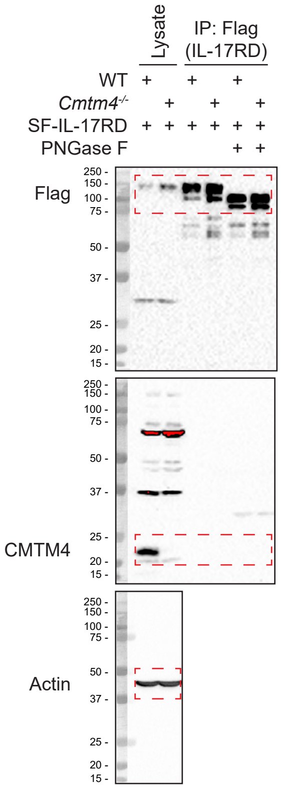

Extended Data Fig. 3i

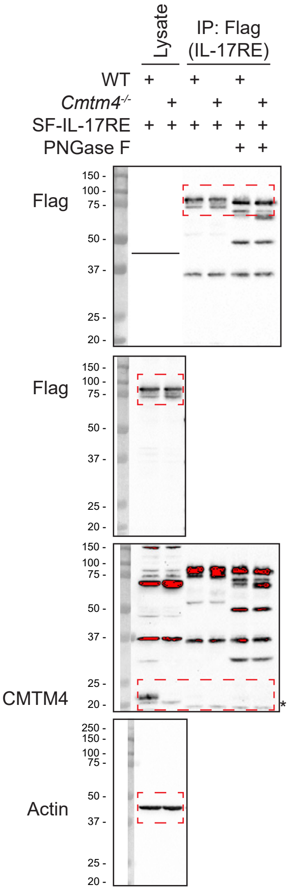

Extended Data Fig. 3j

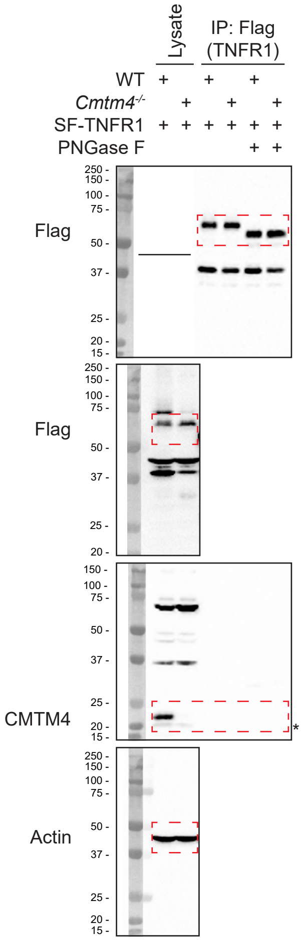

Supplement: Source Data Extended Data Fig. 3 — Unprocessed western blots. [file 41590_2022_1325_MOESM17_ESM.pdf]
